# Investigating Dog Welfare When Interacting with Autistic Children within Canine-Assisted Occupational Therapy Sessions: A Single Case Study

**DOI:** 10.3390/ani13121965

**Published:** 2023-06-12

**Authors:** Jessica Hill, Carlie Driscoll, Judy Cawdell-Smith, Stephen Anderson, Jenny Ziviani

**Affiliations:** 1 School of Health and Rehabilitation Sciences, The University of Queensland, Brisbane, QLD 4072, Australia; carlie.driscoll@uq.edu.au (C.D.); j.ziviani@uq.edu.au (J.Z.); 2 School of Agriculture and Food Sciences, The University of Queensland, Brisbane, QLD 4072, Australia; judycawdellsmith@gmail.com; 3 School of Biomedical Science, The University of Queensland, Brisbane, QLD 4072, Australia; stephen.anderson@uq.edu.au

**Keywords:** human-animal bond, animal welfare, canine-assisted therapy, cortisol, oxytocin

## Abstract

**Simple Summary:**

Recent years have seen growing research highlighting the benefits of human-animal interaction for the health and wellbeing of autistic children. This is particularly so for interaction with dogs. The human-animal bond advocates that the mutual benefits of this interaction for both humans and animals are crucial and that equal consideration be placed on the welfare of the animals. However, limited research is available exploring the impact on the welfare of dogs when interacting with autistic children. This study aimed to assess changes in stress biomarkers including salivary cortisol, alpha amylase, and oxytocin, as well as behaviour observations of a therapy dog involved in canine-assisted therapy sessions with autistic children. Results from this study found that the therapy dog did not experience significant changes in stress indicators on days working with autistic children, compared to days spent at home. This study highlights the need for further research regarding therapy dog welfare when interacting with autistic children.

**Abstract:**

Human-animal bond is defined as the mutually beneficial relationship between humans and animals. Recent years have seen increasing research regarding the benefits of interaction with animals for autistic children. However, there continue to be limited studies exploring the impact of this interaction on the welfare of therapy dogs. As part of a pilot randomised control trial assessing the efficacy of canine-assisted occupational therapy with autistic children, this project assessed welfare markers of the therapy dog involved. A total of twenty-one saliva samples were taken from the therapy dog to assess cortisol, alpha amylase, and oxytocin concentrations at home and throughout the treatment days. Additionally, six hours of therapy session videos were analysed for stress indicators of canine behaviour. No significant differences were found between days spent at home and treatment days for any of the biomarkers or stress indicators. Results suggest that the therapy dog involved did not experience increased stress resulting from interaction with the autistic children throughout the therapy sessions. This study supports the need for further research regarding therapy dog welfare when interacting with autistic children including an increased sample size of therapy dogs and therapists.

## 1. Introduction

Autism spectrum disorder is a lifelong, complex developmental condition consisting of persistent challenges with social communication, restricted interests, and repetitive behaviours [1]. The past two decades have seen an increasing interest in the potential relevance animals play in the lives of autistic children [2]. Specifically, increased research has been conducted exploring the potential benefits of pet ownership, assistance animals, and animal-assisted therapy for autistic children, with dogs being the most frequently researched species [3,4]. It has been noted that, although having reduced motivation to engage with humans, autistic children appear to be motivated to engage with animals [2]. Furthermore, it has been suggested that animals may act as a ‘social lubricant’ for autistic children, acting as a bridge to support the development of relationships with other important people, including family members, friends, teachers and therapists [5,6,7,8,9].

This ‘social lubricant’ effect has been of particular interest to therapists working with autistic children resulting in increased research regarding the influence of animal-assisted therapy for autistic children [4,6,7]. Animal-assisted therapy (AAT) is defined as a planned, structured, documented, goal directed intervention performed by a health professional working within the scope of their profession, in which an animal plays an integral role [10]. For autistic children specifically, animals have been suggested to act as a source of motivation to engage with a therapist, and within therapy sessions [4,6,7,11].

Although the literature displays positive regard for the incorporation of AAT with autistic children, to our knowledge, no current studies exist which explore the impact on animal wellbeing when involved in animal-assisted therapy sessions with autistic children. However, within a study completed by Burrows and colleagues [12], authors explored the welfare of assistance dogs assigned to autistic children. They identified several factors specific to autistic children that may impact the dog’s welfare. Factors identified included behaviours displayed when the children experienced a “meltdown” or displayed aggressive behaviour towards the dog, as well as difficulty interpreting the dog’s body language when not enjoying an interaction, for example, inappropriate touching [12]. The effects of the human-animal bond, in which AAT is based, advocate for the mutual benefits of human-animal interaction [13]. Therefore, with the increasing prevalence of dogs being incorporated into the lives of autistic children [8], it is crucial that researchers and clinicians do not lose sight of the needs of the dogs [14].

When involved in AAT, dogs are expected to complete a thorough assessment prior to beginning work to ensure they are able to act predictably within a familiar environment, as well as appropriately within an unfamiliar environment [15,16]. However, this assessment does not assess the dog’s experience within the workplace. Historically, animal welfare has been assessed by the animal’s ability to participate within the Five Freedoms, including: freedom from hunger or thirst; freedom from discomfort; freedom from pain, injury or disease; freedom from fear and distress, and; freedom to express normal behaviours [17]. It has been suggested that assessing animal welfare can be difficult due to the subjectivity of the handler’s observational assessment. Current research supports the use of salivary biomarkers as a method of assessing animal welfare [18], in particular, the sampling of cortisol and, more recently, alpha amylase (sAA) and oxytocin [15,16,19,20,21,22,23,24].

Cortisol is a commonly used biomarker for measuring human and animal welfare [16,19,25]. An increase in cortisol, a steroid hormone, occurs as a result of the activation of the hypothalamic-pituitary-adrenal-axis in response to stressful stimuli [16,26]. Furthermore, salivary cortisol concentrations have been supported by several studies to be well correlated with plasma cortisol concentrations, reducing the need for invasive blood sampling [25]. However, due to the significant amount of intra-individual and inter-individual variability, as well as difficulty distinguishing between increases in cortisol levels due to stress versus excitement, the sampling of additional biomarkers is required to support the validity and generalisability of results [19,27]. This is particularly relevant if samples are collected at defined time points that might not take such effects into consideration [19]. Finally, it has been suggested that cortisol concentrations may be more reliable when assessing short-term responses to stress rather than across time, again indicating that in order to accurately and reliably assess the welfare of a therapy dog involved in animal-assisted therapy sessions across time, additional measures of assessment are required [27].

Salivary alpha amylase (sAA) is an enzyme released in response to the activation of the sympathetic-adrenomedullary system in canines [28]. There has been previous controversy regarding the use of sAA in canine saliva; however, recent studies have confirmed its presence and, therefore, it has been posited as a possible biomarker for canine stress [28,29,30].

In addition to sAA, in recent times researchers have also started to suggest that the measurement of oxytocin may be used when assessing animal welfare [31,32]. Oxytocin is a neuropeptide produced in the paraventricular and supraoptic nuclei of the hypothalamus and released at the posterior pituitary gland [32]. Known as the ‘bonding’ or ‘love’ hormone, oxytocin is well known for facilitating social attachment in both humans and some non-human species [32]. However, oxytocin also assists to decrease stress by reducing the level of cortisol in the body, lowering blood pressure, and decreasing pain sensation by having an effect in the periaqueductal grey and the spinal cord, as well as, counteracting aggression and arousal levels by impacting the locus caeruleus [32,33].

Although analysis of salivary biomarkers is supported within the literature, many veterinarians and welfare specialists have begun advocating for the continued use of subjective experiences, as it has been suggested that a dog that is simply ‘coping’ may continue to experience poor welfare [17]. Behavioural observations assist in providing researchers with a greater understanding of context, in particular, if the dog is experiencing distress or excitement [19]. Recent stress behaviours explored within therapy dogs involved in animal-assisted therapy have included lip licking, yawning, body shaking, paw lifting, panting, grooming, avoidance, and tail wagging [16,34]. Additionally, it has been suggested that assessment should include the degree in which therapy dogs are able to participate in accordance with their natural behaviours, as this is considered crucial for positive animal welfare [17].

Several ‘safeguards’ have been suggested within the literature to support the protection of therapy dogs participating within animal-assisted therapy sessions [17]. First, therapy dogs are required to be specifically trained and assessed with their handler as a part of a human-canine therapy team [16]. In this assessment, dogs are expected to have a calm and relaxed temperament even when presented with unfamiliar and possibly stressful situations (e.g., rough patting, squealing, throwing objects), as well as respond reliably to both visual and vocal commands [16]. It has been suggested that some training methods in which dogs are forced into certain positions can increase the stress experienced by the dog [16,17]. Additionally, when working with a therapy dog one must acknowledge the removal of its liberty due to being forced into positions such as removal of their ability to walk away, as well as being guided on lead, all of which have been suggested to increase a dog’s stress level during therapy sessions [16,35].

Canine personality and psychological responses may stabilise over time; therefore, the age and experience of the dog has been suggested to have a possible impact on stress levels experienced within animal-assisted therapy sessions [27,35]. This has been supported within the literature with researchers finding reduced cortisol secretion within their older, more experienced therapy dogs when compared to younger, less experienced dogs [35]. Therefore, it has been suggested that therapy dogs reach a minimum age of one to two years prior to starting work [17].

Consideration of client characteristics should also occur, for example, the human participant’s age. Within a 2009 study, Marinelli and colleagues [36] found a significant increase in stress-related behaviours when therapy dogs were involved in sessions with children under the age of 12 years. Additionally, as identified within the 2008 article by Burrows and colleagues [12], client factors such as the child’s behaviour, as well as ability to interpret and respond to the dog’s stress signals, must also be acknowledged.

Timing of the therapy sessions has also demonstrated a possible impact on the stress of the therapy dog, in particular, the number of work hours, frequency of scheduled breaks, human contact time, rest, food and down time breaks [17]. Haubenhofer and Kirchengast [37] found a statistically significant increase in stress-related behaviours for therapy dogs that participated in shorter, more frequent sessions. It was hypothesised by the owners that this was due to the reduced number of breaks the animals received during the short sessions when compared to long sessions [37]. Additionally, King and colleagues [38] found that for the dogs included within their study, cortisol levels increased during their first session, however, appeared to drop by their final session of their day. Authors hypothesised that this might have been due to the dogs habituating to their environment throughout the treating day. Furthermore, researchers have suggested that sessions conducted immediately after transportation may result in an increased cortisol reading [26,35].

Finally, environmental factors have also been found to play a part in increasing stress in working therapy dogs. Ng and colleagues [26] found that therapy dogs working within novel environments demonstrated a significant increase in cortisol levels compared to the baseline of being at home. Furthermore, environmental considerations, such as the room size, restriction of water, and temperature, should also be taken into consideration, with Marinelli and colleagues [36] finding a significant increase in stress-related behaviours within rooms of higher temperatures, as well as smaller, confined conditions. Training of the handler has also been suggested to impact canine welfare with research suggesting that the therapy dog’s handler should complete rigorous training prior to starting work as canine-assisted therapists enabling them to be cognisant of their dogs needs and provide them with safe and predictable experiences [17,34].

Research into the physiological and behavioural responses of dogs within therapy sessions will improve understanding of how canine welfare is impacted by their interaction with autistic children during AAT sessions, hopefully leading to negation of adverse effects on welfare, and contributing to development of appropriate guidelines. Therefore, the aim of this study was to ascertain if participation within canine-assisted therapy sessions with autistic children produced elevated stress levels for a therapy dog, as measured by significant differences in behavioural observations, and salivary cortisol, alpha amylase, and oxytocin, when compared to days spent unemployed at home.

## 2. Materials and Method

Collection procedures followed those outlined within the protocol developed for a pilot randomised control trial exploring the efficacy of canine-assisted occupational therapy for autistic children [39].

### 2.1. Handler-Therapy Dog Team

The therapy dog involved within this study was a three-year-old, desexed, female, standard, gold, wool coat labradoodle, who weighed 25 kg at time of assessment. She had been socialised to the clinic environment from eight weeks of age. The therapy dog had attended general obedience training until the age of twelve months, had completed a five-day intensive handler-canine training and assessment (Therapy Dogs Australia), and had been working as a therapy dog with autistic children for two years prior to commencing the study.

Additionally, the therapy dog involved attended biannual veterinary checks to ensure full physical and emotional wellbeing, as well as ensuring she was up to date with all vaccinations and preventative medications. Medications taken by the therapy dog included Bravecto (one time every three months) for flea and tick prevention, and Drontal (one time every three months) for intestinal worm prevention.

A typical working week for the therapy dog involved two to three days of work with a minimum of one day’s rest between days. Working days would involve a maximum of six fifty-minute sessions with a minimum of ten-minute break between each session, and a minimum of thirty-minute break every three hours. The handler was the owner of the therapy dog (first author), was female, and held a bachelor’s degree in occupational therapy, had completed multiple trainings in animal-assisted therapy (Lead the Way, Therapy Dogs Australia, and International Institute for Animal Assisted Play Therapy, Level 1), had five years’ experience working with autistic children and had been working with her therapy dogs, within a private practice setting, for four years before beginning this study. The therapy dog involved lived with the handler and two other dogs.

### 2.2. Setting

Canine-assisted occupational therapy sessions took place within a private animal-assisted psychology practice, Brisbane, Queensland, specifically designed to facilitate therapy dogs. Two additional therapy dogs were on location working with their handlers, with which the therapy dog was allowed to interact when not within sessions. The room used for all sessions was well known to the therapy dog, as she had been working there for 12 months prior to beginning the study. The room was 3.73 m by 4.31 m square, fully carpeted, and air conditioned. Within the room, there was one couch on which the dog was allowed to sit if invited, a large dog bed, a small toy bag, a small cupboard, and one small filing cabinet. A filled water bowl was placed next to the dog bed at all times. Access to cleaning products, including disinfectant wipes and spray, as well as hand sanitiser, was available at all times and the children were prompted to wash their hands prior to leaving each session.

### 2.3. Study Procedure

The therapy dog was transported to the clinic in a 2018 Holden Colorado dual cab with a customised canopy, including screened windows (allowing the windows to be safely kept open at all times) and a rotary vent located on the roof. Within this enclosure, the therapy dog had access to a bed and toys. Travel to the clinic took approximately 20 min and the dog was provided with a minimum of 30 min rest prior to beginning her first session [35]. The therapy dog saw between three and six clients for 50 min each, per day and was provided with a minimum of thirty-minute breaks between each client.

The therapy dog worked with 11 autistic children aged between four and six years old. Eight children were male and three children were female. The therapy dog worked with the same eleven children across a seven-week period for either weekly or fortnightly appointments [1]. Although each therapy session was tailored to each individual child typical activities and tasks completed by the therapy dog included sitting with the child, playing with a toy with the child and handler, getting a brush, completing ‘tricks’ e.g., rolling a large dice, and sleeping on her bed. Interactions between the therapy dog and the children were guided by the handler who provided education to the children about how the therapy dog liked to be touched e.g., “gentle patting on her back” and “soft brushing”. This was done to reduce the incidence of inappropriate touching. Further, the children were also supported by the handler, who was also a registered occupational therapist to self-regulate throughout the session. On the rare incidence that a child did become dysregulated the therapy dog was trained to respond to the handler’s cues to either stand by her side or move to her mat allowing either the handler or the child’s parent to support the child.

During breaks, the therapy dog was allowed to sleep if desired, and was also taken outside for regular toilet breaks and sniff walks. No moderate to vigorous exercise was completed throughout the treatment day. The therapy dog was provided with treats consisting of carrot slices, home-made dog biscuits and yogurt drops throughout the day, and provided with dinner at 5 p.m. on both working days and rest days.

The therapy dog was provided with a minimum of one rest day between working days which were spent at her home with access to both the inside and outside of the house. On rest days the therapy dog would complete her typical non-working day routine which consisted of a 30-min morning and afternoon walk, sleep and play with two other dogs.

### 2.4. Salivary Sample Collection

**Figure 1:**
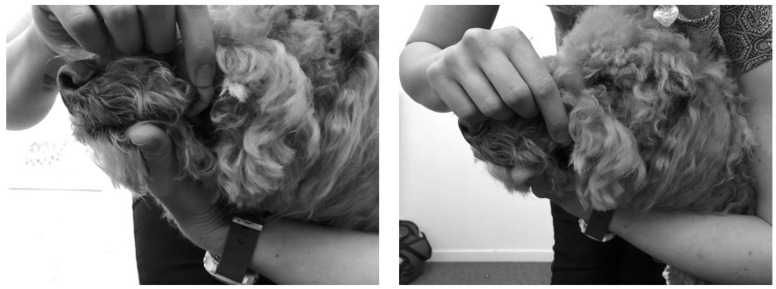
Canine sample collection procedure.

A total of 21 experimental samples were collected from the therapy dog at multiple intervals across a seven-week period of canine-assisted occupational therapy (week one, four, and seven) (Table 1 and Table 2) using SalivaBio Children’s Swabs (Synthetic) [22,40]. No food was provided to the therapy dog for 60 min prior to the sample being taken [22,41]. The swab was placed between the therapy dog’s right mandibular teeth and cheek (Figure 1) by the first author for a total of 90 s, with her mouth gently held shut to avoid swallowing [22,41]. Samples were stored within a sample tube and frozen at −40 °C immediately after extraction, then transferred to a laboratory on ice within a Styrofoam box and frozen at −80 °C within 24 h [41].

A total of three baseline samples were taken from the therapy dog after a typical day at home (week one, four, and seven). Samples were taken at 6:30 p.m. on every occasion, one hour after the first author returned home from work, and a minimum of 30 min after interaction with the first author or others. A food reward was provided immediately after the sample was collected.

A total of 18 treatment samples were taken from the therapy dog during the first and last sessions of a typical treatment day (on Thursday of week one, four, and seven; Table 3). Samples were taken at three intervals: (1) five minutes prior to beginning the therapy session, following a 15-min rest period in which there was no human interaction, and a minimum of 30 min after transportation to the clinic; (2) 20 min after initial interaction with the child within the therapy session, and; (3) 30 min after the end of the therapy session, a period in which there was no human interaction. See Table 1 for a summary of the collection schedule. The therapy dog was highly tolerant of having items placed in her mouth (e.g., toothbrush and medications) and showed no adverse reactions to the samples being taken. A food reward was provided after the second and final sample of the session had been taken to ensure there was a minimum of 60 min between the time food was given and sample collection [21,41].

Salivary cortisol has been found to lag that of plasma cortisol by 20 min resulting in a cortisol peak occurring approximately 20 min after exposure to the stressor and is then maintained for approximately 30 min after that peak [42]. This was taken into consideration when developing the protocol for sample collection.

### 2.5. Salivary Sample Analysis

Salivary biomarkers were determined using commercial kits; sAA activity (sAA kinetic enzyme assay kit, 1-1905, Salimetrics, State College, CA, USA), salivary cortisol (enyme immunoassay kit, 1-3002 Salimetrics, State College, CA, USA), and salivary oxytocin (Oxytocin ELISA, 500440, Cayman Chemical, Ann Arbor, M, USA). Salivary samples were diluted 1:100, 1:10 and 1:25 in assay diluent for each assay, respectively. Saliva samples were not extracted prior to oxytocin assay, as previously validated in another dog study [22]. All assays were performed according to the manufacturer’s protocols.

### 2.6. Behavioural Observations

In addition to the salivary biomarker analyses, a total of six hours of session video were analysed for canine behaviour. Videoing began a minimum of 1 min before the child entered the room and was stopped 1 min after the child exited. Due to the limited number of published assessment tools suitable for the purpose, the *Behavioural Instrument for the Assessment of Dog Well-Being Before/During/After Therapy Sessions* was chosen [43]. This checklist consists of ten items assessing the therapy dog’s aggression, fear/anxiety/stress, excitability, interaction with people, interaction with dogs, obedience, tiredness, reactivity, anticipation, and a final score factor if any item scored above a three. Each item is scored between a zero to three depending on the severity of the behaviour, for example, for the item assessing fear/anxiety/stress, zero would be scored if there were “no sign of fear, anxiety or stress”, one if the dog/s “lick lips/nose, excessive salivation, pawing, tail between legs, no eye-contact”, two if the dog was observed “crouching both standing and sitting, whining, shaking, restless, agitated, yawning” and three if the dog was “cowering, whale eye, attempting to escape from the situation” [43] (p. 371). Scores for each item are then added up to provide a total. Total scores of 1–10 suggest that the well-being of the therapy dog is “generally acceptable”, 11–15 suggests the well-being “may start to be affected”, 16–24 suggests “well-being impact is more serious”, and ≥25 suggests the therapy dog’s “well-being is severely affected” [43] (p. 371). Videos were taken during the same sessions as the treatment samples (i.e., the first and last sessions of the sample collection days), by one Go-Pro camera placed within the corner of the clinic room allowing the therapy dog to be in view at all times.

Although useful in understanding the context of the therapy dog’s interactions, the interpretation of stress-related behaviours in dogs has been contentious within the literature [19]. Therefore, to ensure the integrity of the results, videos were scored by two observers, one of whom was blinded to the aims of the study and the other whom was the first author and owner of the therapy dog. This was done as the first author knew her therapy dog well and understood the context of the therapy session. Both observers scored all six hours of video independent of each other to enable assessment of inter-rater reliability. Observer 1 practiced as an occupational therapist and had completed additional training in animal-assisted therapy and canine behaviour.

### 2.7. Statistical Analysis

Analysis was completed using the Statistical Package for the Social Sciences (SPSS) version 25 [44]. A one-way ANOVA was used to determine any significant difference between the saliva biomarkers from days spent at home compared to treatment days.

## 3. Results

### 3.1. Saliva Biomarkers

**Figure 2:**
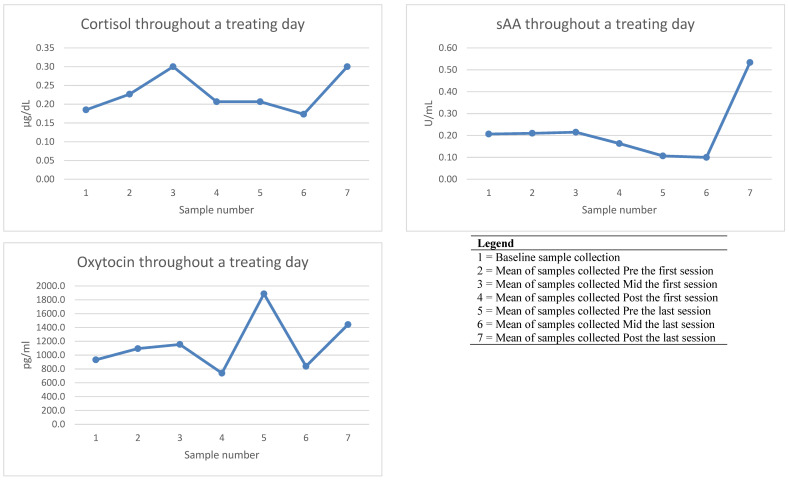
Trend of saliva biomarkers from baseline to after last treatment session (means of three collection day).

A total of twenty-one salivary samples were collected. Of the collected samples, twenty-one yielded sufficient saliva for cortisol analysis, nineteen yielded sufficient saliva for alpha amylase, and twenty-one yielded sufficient saliva for oxytocin analysis (Table 4). One baseline cortisol reading was identified as an outlier and, therefore, was not included within the final analysis. The mean results for salivary biomarkers (averaged across the three collection days) are outlined in Figure 2.

Cortisol concentrations within the therapy days ranged from 0.14–0.54 µg/dL. Mean cortisol concentrations across the collection days were 0.19 µg/dL (baseline at home), 0.23 µg/dL (pre first session of the day), 0.30 µg/dL (mid first session of the day), 0.21 µg/dL (post first session of the day), 0.21 µg/dL (pre last session of the day), 0.17 µg/dL (mid last session of the day), and 0.30 µg/dL (post last session of the day). Results showed that there was a small increase in salivary cortisol concentrations from the baseline (n = 2, 0.19 ± 0.03) across all therapy days and time points (n = 15, 0.22 ± 0.07), albeit the increase was not significant.

sAA activity levels in the therapy days ranged from 0.03–0.82 U/mL. Mean results for alpha amylase across the collection days included 0.21 U/mL (baseline at home), 0.21 U/mL (pre first session of the day), 0.22 U/mL (mid first session of the day), 0.16 U/mL (post first session of the day), 0.11 U/mL (pre last session of the day), 0.10 U/mL (mid last session of the day) and 0.30 U/mL (post last session of the day). Overall, results showed that there was a non-significant (*p* > 0.05) increase in salivary alpha amylase activity from the baseline (n = 3, 0.21 ± 0.16) to across all therapy days and timepoints (n = 16, 0.23 ± 0.19).

Salivary oxytocin concentrations in therapy days ranged from 272–2309 pg/mL, with average of 932 pg/mL (baseline at home), 1095 pg/mL (pre first session of the day), 1155 pg/mL (mid first session of the day), 740 pg/mL (post first session of the day), 1887 pg/mL (pre last session of the day), 839 pg/mL (mid last session of the day), and 1443 pg/mL (post last session of the day). Results showed that there was an increase in oxytocin from baseline concentrations (n = 3, 932.11 ± 225.27) across all therapy days and timepoints (n = 18, 1192.86 ± 700.83), although high variability resulted in no significant differences (*p* >0.05).

### 3.2. Behavioural Observations

High levels of inter-rater reliability were found when using the *Behavioural instrument for the assessment of dog well-being before/during/after therapy sessions* [43] with coders achieving 90.38% agreement across ten items. The lowest total score across all six sessions was zero, with the highest score being three. All scores were within the 0–10 range meaning the “well-being is generally acceptable—The dog shows no clear sign of being affected by her/his involvement in the therapy session and no special measures need to be taken” [43] (p. 317). Items on which the therapy dog scored higher than a zero included ‘fear/anxiety/stress’ and ‘excitability.’ Stress-related behaviours observed included lip lick, yawn, high ears, and a shake off. Of these stress behaviours, it was identified that 26% occurred when playing with a toy with a child, 19% occurred when inconsistent instructions were provided from either the handler or the child, 19% occurred when waiting for a treat, 17% occurred immediately after finishing a treat, 14% occurred when the dog was waiting for instructions, and 5% occurred during an unpleasant interaction with a child, e.g., rough patting on the head.

The greatest difference observed between coders was within the item ‘fear/anxiety/stress’ with coder 2 scoring higher across four sessions and coder 1 scoring higher in one session. It was discussed between coders that coder 2 recorded 1 if even one stress signal was observed throughout the session (e.g., if the therapy dog only lip licked one time) whilst coder 1 made a subjective decision if the therapy dog had displayed enough stress behaviours to meet the criteria of a 1. This led to the discrepancy between coders.

## 4. Discussion

Over the past 20 years, there has been an increased interest in the influence of animals in the lives of autistic children [2]. However, there remains limited information regarding the impact to animals during these interactions. To our knowledge, this is the first study completed assessing the welfare of a therapy dog when working with autistic children within animal-assisted therapy sessions. When following the protocol developed for this study [39], results collected from the saliva samples, as well as the behavioural observations, appeared to show that the therapy dog involved did not experience significant stress when interacting with autistic children during AAT sessions. It should be noted that this study focused on one, three-year-old, female, labradoodle who had three years’ experience working with autistic children. It should also be acknowledged that the therapy dog’s handler was a registered occupational therapist with additional training in animal-assisted therapy and had five years clinical experience working with autistic children. This experience had provided the handler with the knowledge and skills to appropriately respond to the needs of the children and therapy dog throughout the therapy sessions.

Results from the dog’s saliva indicated no significant increase in cortisol, alpha amylase or oxytocin during the treatment day when compared to a typical day spent at home (baseline). Additionally, in a systematic review and meta-analysis establishing normal values and ranges of canine salivary cortisol concentration and the impact on physical and environmental factors completed by Cobb and colleagues [27], the authors identified mean salivary cortisol concentrations of dogs to be 0.45 µg/dL. The highest mean concentration in our current study was 0.30 µg/dL supporting low salivary cortisol concentrations and stress, methodological differences notwithstanding.

Scores obtained from the Behavioural instrument for the assessment of dog well-being before/during/after therapy sessions showed that the therapy dog’s well-being was “generally acceptable” as she demonstrated no clear signs of being affected by her involvement in the therapy sessions, with no special measures needing to be taken [43].

During the therapy sessions, a positive trend in salivary biomarkers was observed from baseline (at home) to post-treatment sessions (the end of working days) although this was not significant for any biomarker. Specifically, cortisol peaked in the middle of the first session of the day and again after the last session. sAA remained relatively stable, at or below baseline, until peaking after the last session. Oxytocin peaked before and after the last session. The trend in cortisol concentrations is consistent with previous research on salivary cortisol and behavioural observations of therapy dogs participating in 60-min, canine-assisted activity sessions [26,38] and 60-min group canine-assisted therapy sessions [35], which demonstrated increases in cortisol within the first session, before dropping by the final session. The trend for increased cortisol and sAA at the end of the last session in the current study could be due to numerous factors, including an accumulative stress effect at the end of the day, anticipation about returning home, or anticipation before the dog’s dinner. It should be noted the final sample was taken at the dog’s typical dinner mealtime. The peak in oxytocin levels immediately before the last session of the day is difficult to explain and despite reviewing video footage during this time period, the first author/handler is unable to suggest a definitive reason for any cortisol increase. Perhaps, the dog was aware that her workday would soon be coming to a close.

The peaks in cortisol and sAA after the final session of working days (positive indicators of stress) combined with the peak in oxytocin (often perceived as a negative indicator of stress) highlights the interpretative difficulties that arise when attempting to analyse stress responses. In fact, oxytocin does not simply downregulate the action of the HPA axis. It can, actually, be released not just from positive events, but from intense stressors also, resulting in transient activation of the HPA axis and sympathetic nervous system. High levels in the blood can be detected after both positive and negative events.

Past studies have demonstrated conflicting views on the welfare of dogs involved in animal-assisted therapy with some finding no difference in stress at baseline compared to treating sessions [16,26], whilst some have found an increase in salivary cortisol levels of dogs participating in therapy sessions compared to time spent at home [37,38]. Within the current research, several conditions have been suggested to facilitate welfare in dogs participating in animal-assisted therapy which were followed within the present study.

When exploring canine characteristics, it has been suggested that age can have an impact with research suggesting older dogs with more experience display fewer stress signals compared to younger dogs [17,27,35]. The therapy dog included within this study was three years old at the time of data collection and had two years previous experience working with autistic children within a private practice setting. Additionally, the therapy dog incorporated was a neutered female with previous research suggesting that neutered females release reduced levels of cortisol compared to intact females [27]. Breed, weight, coat colour and type have been found to have no influence on canine stress or welfare [27].

When working with their handler, it has been identified that dogs working with same gendered handlers demonstrated a positive impact on canine welfare when in animal-assisted therapy sessions [15,27]. Both the dog and the handler involved within this study were female. Additionally, the handler’s ability to be aware and respond to the therapy dogs needs within the session (e.g., monitor the environment and interactions) have been shown to improve the welfare of dogs included within therapy sessions. The handler included within the present study had additional training in animal-assisted therapy, as well as in canine behaviour, allowing her to therapeutically respond to her dog’s needs within the session [17,34].

Environmental factors have been suggested to have an impact on dogs working within a therapy position, such as therapy dogs working within a novel environment [19,26,36]. The therapy dog had been socialised to the clinic environment from eight weeks of age and had been working two to three days per week within the specific clinic room in which the study was conducted for twelve months prior to commencement. Studies have shown that clinic room size and temperature impact the cortisol secretion and stress behaviours of therapy dogs [19,36]. Additionally, frequent changes in floor surfaces (e.g., moving from carpet, to tiles, to grass) has been suggested to increase the stress of therapy dogs [19]. The clinic space used within this study was 3.73 m by 4.31 metres square and fully carpeted. The room was airconditioned at all times and was set on 18–25 °C depending on the outside temperature as the present study was conducted during summer in Queensland, Australia.

The timing of sessions has been suggested to have an impact on canine welfare, with Haubenhofer and Kirchengast [37] finding that shorter and more frequent sessions appeared to increase stress-related behaviours of therapy dogs due to the reduced number of breaks the dogs received during the shorter sessions. Within the present study, although lasting for a total of 50–60 min, the therapy dog was actively involved (interacting with handler and/or child) for a maximum of 15 min per session. For the remainder of the session, the therapy dog was free to lie down and rest/sleep. Additionally, the therapy dog was provided with regular breaks in between sessions, where she was free to interact with the other dogs within the clinic and was taken for regular walks outside [17].

Finally, the therapy dog’s ability to access water freely, as well as maintain its liberty, has also been identified within the current literature as important to maintaining canine welfare [16,17,35]. The therapy dog included within the present study was off lead at all times when within the clinic, allowing her to move away from unwanted interactions. Additionally, a clean bowl of water was located in a safe location within the room throughout the treating day.

### Limitations

A significant limitation of this study was the inclusion of only one dog. As the present study was part of a randomized control trial for canine-assisted occupational therapy for autistic children, it was only possible to assess the saliva biomarkers and behaviours of a single therapy dog. Further research is required with multiple canine and handler teams following the same protocol [39] in order to generalise the results found within this study.

A second constraint was the limited number of physiological biomarkers assessed within this study. It was initially attempted to assess the heart rate (HR) of the therapy dog, however, the handler experienced great difficulty securing the HR monitor onto the chest of the therapy dog without inducing further stress. The HR monitor was to be secured around the therapy dog’s chest with the monitor placed directly on her heart. As the therapy dog was off lead and frequently active throughout the day (e.g., lying on her bed, getting up to perform a trick, jumping up on the couch, playing with a toy), the HR monitor would frequently slip off. The idea of applying a cohesive medical vet wrap around the chest strap was discussed, however, when tested it was observed to increase the stress experienced by the therapy dog. Therefore, it was decided that HR would not be monitored within this study, but clearly would be a good marker of stress activation of the autonomic nervous system.

The total number of saliva samples collected over the treatment period was identified as a limitation. Although not statistically significant, there was a positive trend observed within all biomarkers from baseline (home) to treatment (the end of the working day). If saliva samples had been taken over an increased number of time points or over a greater number of days, a significant impact may have been observed. Furthermore, finer level comparisons, such as averaged baseline levels versus levels obtained for the average of pre-therapy, mid-therapy, or post-therapy time points separately, could not be made due to insufficient sample size.

The difficulty obtaining enough saliva to assess cortisol, alpha amylase and oxytocin was identified as a limitation. It was identified that a minimum of 100 µL would be needed to complete the three assays [21]. In a trial to assess for the most effective position within the dog’s mouth, it was identified that when collecting saliva from the dog’s left cheek approximately 30 µL was extracted, 60 µL was extracted when placing the swab at the front of the therapy dog’s mouth under her tongue, and approximately 100 µL was extracted when placed within her right cheek. Difficulty extracting the correct amount of saliva from the salivette resulted in missing data. Finally, future studies would also benefit by comparing diurnal cortisol and oxytocin measurements on control days versus therapy days.

## 5. Conclusions

Over the past decade, there has been an increased interest in the impact of animals in the lives of autistic children; however, there remains limited research into the impact of these interactions on the wellbeing of the animals. Additionally, for animals incorporated into animal-assisted therapy specifically, there continues to be limited available guidelines outlining the safe and ethical implementation of canine-assisted therapy which takes into consideration the welfare of both the humans and therapy dogs. When following the protocol outlined within this study, the therapy dog was found to experience minimal stress; however, further rigorous research is required on a larger sample size of dogs to assist with generalisation to therapy dogs working with autistic children. This study highlights the importance of further investigation into the impact of participation in canine-assisted therapy for the therapy dogs involved.

## Figures and Tables

**Table 1:** Canine baseline saliva sample collection schedule.

Baseline Sample	
8:30–5:00 p.m.	Typical day at home
5:00 p.m.–5:30 p.m.	Handler arrives home and given dinner
5:30 p.m.–6:30 p.m.	Rest
6:30 p.m.–6:35 p.m.	Baseline sample taken

**Table 2:** Canine treatment saliva sample collection schedule.

Treatment Sample	
8:30 a.m.–9:25 a.m.	Arrival at clinic—Rest
9:30 a.m.–9:35 a.m.	Pre-therapy sample taken
9:35 a.m.–10:30 a.m.	Client 1 (sample taken 20 min after active interaction with child)
10:30 a.m.–11:00 a.m.	Rest
11:00 a.m.–11:05 a.m.	Post-therapy sample taken
11:05 a.m.–12:00 p.m.	Client 2 (no sample taken)
12:00 p.m.–12:30 p.m.	Rest
12:30 p.m.–1:30 p.m.	Client 3 (no sample taken)
1:30 p.m.–2:00 p.m.	Rest
2:00 p.m.–3:00 p.m.	Client 4 (no sample taken)
3:00 p.m.–3:30 p.m.	Rest
3:30 p.m.–3:35 p.m.	Pre-therapy sample taken
3:35 p.m.–4:30 p.m.	Client 5 (sample taken 20 min after active interaction with child)
4:30 p.m.–4:55 p.m.	Rest
4:55 p.m.–5:00 p.m.	Post-therapy sample taken (followed by dinner)

**Table 3:** Canine saliva sample collection schedule.

Week	Day	Session	Number of Samples
1	Wednesday	Baseline	1
1	Thursday	First	3
1	Thursday	Last	3
4	Wednesday	Baseline	1
4	Thursday	First	3
4	Thursday	Last	3
7	Wednesday	Baseline	1
7	Thursday	First	3
7	Thursday	Last	3

**Table 4:** Canine saliva biomarker results.

Week	Collection Description	Time	Cortisol (µg/dL)	A-Amylase (U/mL)	Oxytocin (pg/mL)
Week 1	Baseline	6:30 p.m.	0.22	0.39	995.8
	Pre first session	9:30 a.m.	0.22	0.20	1397.7
	Mid first session	9:55 a.m.	0.20	0.23	2151.2
	Post first session	11:00 a.m.	0.19	0.20	1118.7
	Pre last session	3:30 p.m.	0.24	0.03	2309.9
	Mid last session	3:55 p.m.	0.14	0.10	1835.7
	Post last session	5:00 p.m.	0.44	0.82	2018.1
Week 4	Baseline	6:30 p.m.	0.15	0.13	1118.7
	Pre first session	9:30 a.m.	0.28	0.23	443.3
	Mid first session	9:55 a.m.	0.54	Missing data	316.7
	Post first session	11:00 a.m.	0.15	0.16	271.6
	Pre last session	3:30 p.m.	0.16	0.16	1189.5
	Mid last session	3:55 p.m.	0.16	Missing data	823.8
	Post last session	5:00 p.m.	0.24	0.52	325.0
Week 7	Baseline	6:30 p.m.	1.09 (outlier)	0.10	681.9
	Pre first session	9:30 a.m.	0.18	0.20	1442.6
	Mid first session	9:55 a.m.	0.16	0.20	804.0
	Post first session	11:00 a.m.	0.28	0.13	996.6
	Pre last session	3:30 p.m.	0.22	0.13	2161.4
	Mid last session	3:55 p.m.	0.22	0.10	355.5
	Post last session	5:00 p.m.	0.22	0.26	1485.9

## Data Availability

The data presented in this study are available on request from the corresponding author. The data are not publicly available due to privacy and consent requirements.
